# Three-dimensional unsupervised probabilistic pose reconstruction (3D-UPPER) for freely moving animals

**DOI:** 10.1038/s41598-022-25087-4

**Published:** 2023-01-04

**Authors:** Aghileh S. Ebrahimi, Patrycja Orlowska-Feuer, Qian Huang, Antonio G. Zippo, Franck P. Martial, Rasmus S. Petersen, Riccardo Storchi

**Affiliations:** 1 Division of Neuroscience, School of Biological Science, Faculty of Biology, Medicine and Health, University of Manchester, Manchester, UK; 2 Institute of Neuroscience, Consiglio Nazionale delle Ricerche, Milan, Italy

**Keywords:** Computational models, Machine learning, Computer modelling, Computer science, Software, Sensorimotor processing

## Abstract

A key step in understanding animal behaviour relies in the ability to quantify poses and movements. Methods to track body landmarks in 2D have made great progress over the last few years but accurate 3D reconstruction of freely moving animals still represents a challenge. To address this challenge here we develop the 3D-UPPER algorithm, which is fully automated, requires no a priori knowledge of the properties of the body and can also be applied to 2D data. We find that 3D-UPPER reduces by $>10$ fold the error in 3D reconstruction of mouse body during freely moving behaviour compared with the traditional triangulation of 2D data. To achieve that, 3D-UPPER performs an unsupervised estimation of a Statistical Shape Model (SSM) and uses this model to constrain the viable 3D coordinates. We show, by using simulated data, that our SSM estimator is robust even in datasets containing up to 50% of poses with outliers and/or missing data. In simulated and real data SSM estimation converges rapidly, capturing behaviourally relevant changes in body shape associated with exploratory behaviours (e.g. with rearing and changes in body orientation). Altogether 3D-UPPER represents a simple tool to minimise errors in 3D reconstruction while capturing meaningful behavioural parameters.

## Introduction

High quality reconstruction of poses and movements is fundamental to quantify animal and human behaviours. This is a key step in many fields of neuroscience seeking to link behaviour with brain functional and anatomical circuitries^1–4^. Recent (< 5 years) advances in Computer Vision finally enabled to reliably track visible body parts obtaining performances comparable to human observers^5–11^. These algorithms have been immediately adopted to quantify such diverse behaviours as locomotion, skilled movements, hunting, escape, courtship and aggression^12,13^.

**Figure 1:**
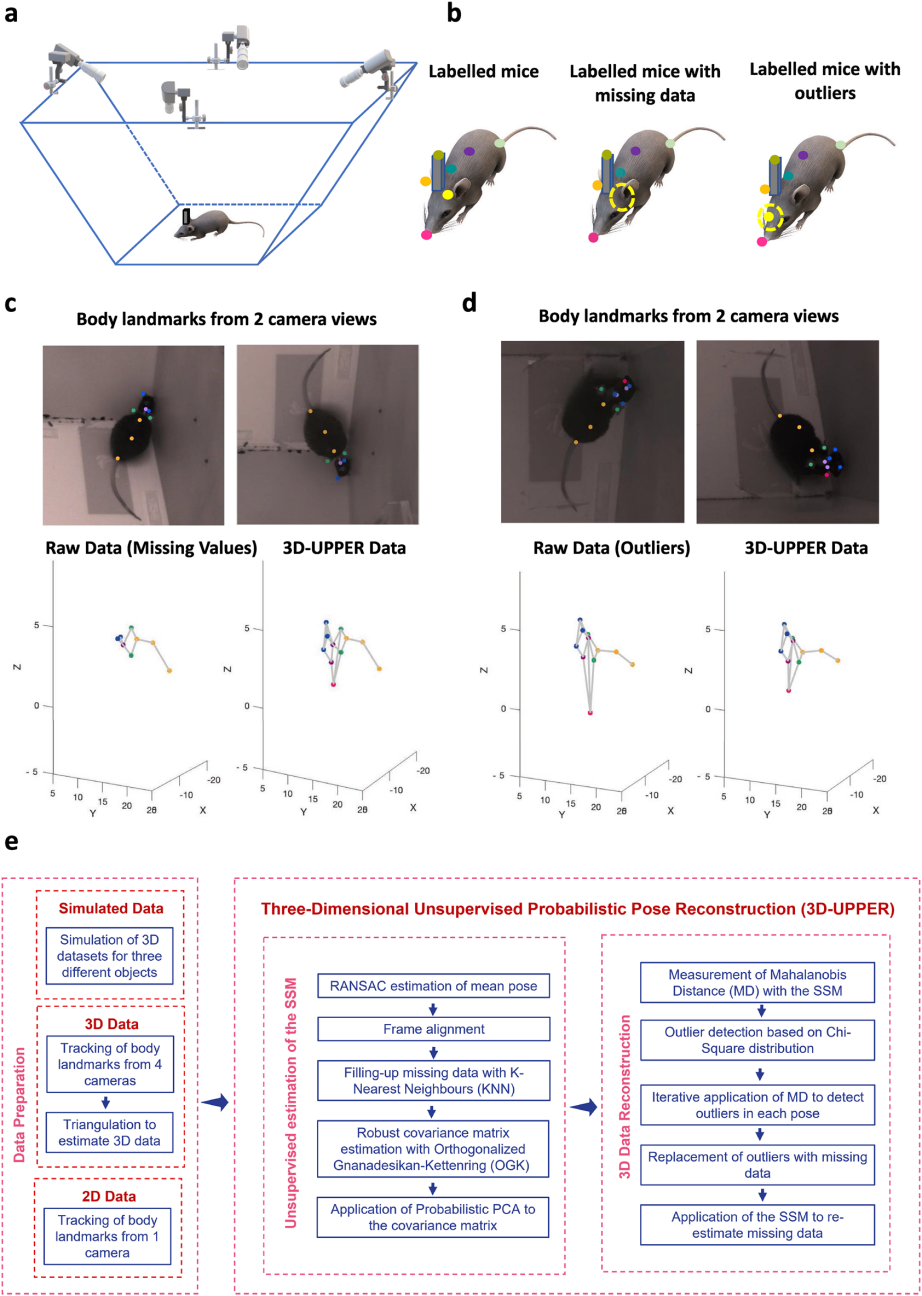
(**a**) A schematic of the behavioural arena, with four cameras positioned above the arena. (**b**) Drawings of a correctly labelled animal (left), of an animal missing labelling on the left ear (middle) and of an animal in which left ear labelling has been erroneously located on the snout (right). (**c**) Automatic labelling of freely moving animals (top), “raw” 3D data after triangulation (bottom left) and recovered 3D reconstruction with 3D-UPPER (bottom right). Since some labels are missing from automatic labelling, the raw 3D reconstruction is contaminated by missing data. The 3D-UPPER algorithm is able to re-estimate the missing data. (**d**) Same as panel c but here the nose label is incorrectly located introducing an outlier in the 3D reconstruction. The 3D-UPPER algorithm can detect the outlier and re-estimate its position. (**e**) Flow chart of data preparation and the 3D-UPPER algorithm.

Despite such remarkable progress, the reconstruction of freely moving animals in which body parts are occluded from camera views still represents an open problem. Tracking occluded body parts results in missing data and outliers as reported by several authors^9,10,14,15^ (Fig. 1a,b). The problem is exacerbated when images from multiple views are combined to generate a 3D reconstruction since erroneous localization from individual views can cause dramatic errors when data are triangulated. Since occlusions are common in environments in which animals can freely express their full motor repertoires (e.g. in an open field arena), solving this problem is key to enable the study of natural unconstrained behaviours.

This paper addresses the problem of correcting 3D datasets contaminated by outliers and missing values via a novel fully automated algorithm: 3D Unsupervised Probabilistic Pose Reconstruction (3D-UPPER). We assume that an existing tracking software (e.g. DeepLabCut^11^) has been applied to video recordings and body landmarks have been triangulated to generate initial 3D datasets. The 3D-UPPER algorithm receives as input such 3D datasets and returns a new 3D dataset in which outliers and missing data have been detected and re-estimated (see e.g. Fig. 1c,d).

The idea behind our algorithm is to use the knowledge of the geometrical relations between body parts to re-estimate incorrectly reconstructed (or missing) data points. Such relations arise from biomechanical constraints of postures and movements. To capture them we resort to using Statistical Shape Models (SSM), a popular family of models in medical imaging^16,17^ that can fit deformable objects and bodies. Those models are typically estimated in a supervised fashion from manually annotated data and this procedure can be biased and time-consuming^14,17,18^. Therefore, we first developed a robust unsupervised SSM estimator that does not require annotated data and can handle 3D datasets contaminated by large numbers of outliers and missing data (Fig. 1e**,** Unsupervised SSM Estimation). Secondly, we developed a method to correct outliers (and/or missing data) from the same datasets by using the previously estimated SSM (Fig. 1e**,** 3D Data Reconstruction). Altogether, SSM estimation and outlier/missing data correction constitute the 3D-UPPER algorithm.

We first applied 3D-UPPER to simulated datasets, since this enabled us to assess its performances against ground-truth data. We then applied 3D-UPPER to a 3D dataset of freely moving mouse behaviour and compared its performances to those obtained by manual labelling. Finally, we showed that our methods can also be applied to 2D data (2D-UPPER). Overall, the results indicate that 3D-UPPER provides substantial improvements in accuracy $>10$ fold over 3D reconstructions obtained by simply triangulating 3D data even when data are contaminated by large fraction of outliers (up to 50%). Its 2D version, 2D-UPPER did not significantly improved body reconstruction but its associated SSM enabled the capture of behaviourally relevant actions such as rearing or body turning.

## Results

### Overview of the 3D-UPPER algorithm

The 3D-UPPER algorithm is divided into two parts. In the first part, 3D-UPPER estimates an Unsupervised Statistical Shape Model (SSM) that captures the mean body shape of the target body and the main directions in which the body shape changes. The description of this part of the algorithm is provided in the following sections: Definition of a statistical shape model, Robust estimation of the mean pose, Robust estimation of the covariance matrix. The full flow chart for unsupervised estimation of a Statistical Shape Model is shown in (Fig. 1e, Unsupervised SSM Estimation).

In the second part, 3D-UPPER applies the SSM first to automatically detect outliers in the data and finally to obtain a robust 3D reconstruction by replacing outliers and missing data. The description of this part of the algorithm is provided in the following sections: Outlier detection, Robust 3D reconstruction. The full flow chart for outlier detection and robust 3D reconstruction is shown in (Fig. 1e, right panel).

After describing the 3D-UPPER algorithm, we validate its performance on a dataset of simulated data and on 3D and 2D datasets of freely moving mice. The results are reported in the following sections: Validation of 3D-UPPER on simulated data, Validation of 3D-UPPER on 3D freely moving data, Validation of 3D-UPPER on 2D freely moving data.

### Definition of a statistical shape model

We assume that all 3D poses of a target body can be described by a mean pose and a linear combination of orthonormal vectors that captures non-rigid changes in the body shape. We define each three-dimensional pose $X$, containing ${N}_{bp}$ body points, through the following equation:

1 $$X= \left({\rm M}+ \sum_{j=1}^{{N}_{ep}}{P}_{j}{b}_{j}\right)R+ T+\mathrm{E}$$

where ${\rm M}$ is a ${N}_{bp}\times 3$ matrix representing the mean pose, ${P}_{j}$ is a ${N}_{bp}\times 3$ matrix representing one of the ${N}_{ep}$ eigenposes, ${b}_{j}$ is scalar and represents the shape parameters, $R$ is a $3 \times 3$ rotation matrix, $T$ is an ${N}_{bp} \times 3$ translation matrix and $E$ is a ${N}_{bp} \times 3$ matrix representing the gaussian noise associated with the experimental measurement of the body points coordinates.

Given biomechanical constraints on real bodies, changes in body shape have a low ${N}_{ep}$- dimensional structure embedded in a high $3{N}_{bp}$- dimensional space, where the remaining dimensions represent noise in the measurements and ${N}_{ep}\le 3{N}_{bp}$.

Then, in absence of rotations and translations, the pose distribution can be described by a multivariate Gaussian distribution as follows:

2 $$N\left({\varvec{x}}|{\varvec{\mu}}, \widetilde{C}\right)=\frac{1}{\sqrt{2{\pi }^{3Np}\left|\widetilde{C}\right|}}\mathrm{exp}(-\frac{1}{2}{({\varvec{x}}-{\varvec{\mu}})}^{T}{\widetilde{C}}^{-1}({\varvec{x}}-{\varvec{\mu}}))$$

where ${\varvec{x}}$ and ${\varvec{\mu}}$ are the vectorised versions of a pose $X$ and the mean pose ${\rm M}$ from Eq. (1) and the covariance matrix $\widetilde{C}$ captures both biomechanical constraints applied to changes in body shape and the measurement noise. The matrix $\widetilde{C}$ can be further decomposed as $\widetilde{C}=V\widetilde{\Sigma }{V}^{T}$ where $\Sigma$ is a $3{N}_{bp} \times 3{N}_{bp}$ diagonal matrix of eigenvalues and the columns of the matrix $V= \left[{v}_{1}, \dots , {v}_{3{N}_{bp}}\right]$ represent the eigenvectors.

In order to isolate biomechanical constraints from measurement noise we can apply a Probabilistic Principal Component Analysis^19^. Thus, the eigenvectors associated with the largest ${N}_{ep}$ eigenvalues correspond to the eigenposes while the remaining $\left(3{N}_{bp}- {N}_{ep}\right)$ eigenvalues are all equalled to ${\sigma }^{2}$ to capture the isotropic noise in the measurement.

According to these definitions, estimating the Statistical Shape Model corresponds to extracting ${\varvec{\mu}}$ and $\widetilde{\Sigma }$ and $V$ from the data. Such estimates are straightforward in a dataset in which data are not contaminated by noise, outliers and missing values and all poses are aligned ($R= T=0$). However, in any realistic dataset, all these issues are present. Below we describe the methodological steps to address them.

### Robust estimation of the mean pose

To obtain a robust estimate of the mean pose ${\varvec{\mu}}$ Eq. (2) we combined the RANdom SAmple Consensus algorithm (RANSAC)^20^ with Procrustes Superimposition^21^.

First, we had to identify a good reference pose to which we will align the other poses. To do that, a pose without missing values was randomly chosen as reference and a random subsample (50%) of the full dataset of poses was used to score the quality of such references. Second, all poses in the subsample were aligned to the reference pose by applying Procrustes Superimposition. Finally, the reference pose was scored by counting the “neighbour poses”, i.e. the number of poses whose Euclidean distance to the reference was below a predefined threshold. These steps were repeated 100 times. The reference pose with the highest score was then selected. Finally, estimation of the mean pose was obtained by averaging all the poses aligned with the chosen reference pose. In this way, we discounted the possibility of a poor choice of reference pose for aligning the data.

The threshold for defining the neighbourhood was automatically tuned before running the core algorithm as the first quartile of pairwise Euclidean distance between pairs of aligned poses in full dataset.

### Robust estimation of the eigenposes

Estimation of the eigenposes relies on an accurate estimation of the covariance matrix C Eq. (2). We first developed an algorithm based on K-nearest neighbours to fill up missing values in the dataset (see “Methods”). After completing the SSM estimation these filled-up values will be re-assessed according to the well-defined SSM confidence intervals, and re-estimated when they fall outside such intervals (see sections “Outliers detection” and “Reconstruction of missing data”). However, at this stage, an initial fill-up procedure is required to be able to apply the Orthogonalized Gnanadesikan-Kettenring (OGK) covariance matrix estimation^22^. The matrix $C$ obtained with OGK has two important properties: it is robust to the presence of outliers, making it ideal for our purposes, and its calculation is deterministic and fast^22^. However, the matrix $C$ cannot be reliably used to capture the pose distribution Eq. (2) since the OGK estimator does not guarantee to return a full-rank, invertible matrix.

To eliminate potential singularities in $C$, we adopted the framework of Probabilistic Principal Component Analysis^19^. We first diagonalised the covariance matrix of $C$ so that $C=V\Sigma {V}^{T}$. We then recalculated a new covariance matrix $\widetilde{C}=V\widetilde{\Sigma }{V}^{T}$ where $\widetilde{\Sigma }$ is a modified diagonal matrix in which the smallest $3{N}_{p}-r$ eigenvalues were replaced by their average value ${\sigma }^{2}$ calculated as:

3 $${\sigma }^{2}=\frac{1}{3{N}_{p}-r}\sum_{i=r+1}^{3Np}{\sigma }_{i}^{2}$$

and ${\sigma }_{i}^{2}$ represent the *i*th eigenvalue from the diagonal matrix $\Sigma$. Since ${\sigma }^{2}$ replaced the smallest eigenvalues, for a reasonable choice of $r$ we could always obtain a full rank covariance matrix.

In line with our definition of a Statistical Shape Model (see “Definition of a statistical shape model” section) the first $r$ eigenvectors, the eigenposes, capture meaningful changes in body shape while ${\sigma }^{2}$ captures the isotropic noise in a measurement.

### Detection of outliers

After the estimation of a statistical shape model we can use this model to detect outliers in the data. Our procedure for outliers detection works in two steps. In the first step we assess the possibility that a given pose contains outliers. In the second step we identify the specific body points associated with outliers within such pose. Upon identification, such body points are deleted and re-estimated. The re-estimation algorithm is described in the following section (see “Reconstruction of missing data”).

First, in order to assess the possibility that a given pose contains outliers, we calculated the Mahalanobis distance ($MD$)^23^ between a given pose ${\varvec{x}}=[{x}_{1}, \dots ,{x}_{3Np}]$ and the statistical shape model (${\varvec{\mu}},\widetilde{C}$):

4 $$MD= \sqrt{{\left({\varvec{x}}-{\varvec{\mu}}\right)}^{{\varvec{T}}}{\widetilde{C}}^{-1}({\varvec{x}}-{\varvec{\mu}})}$$

Any pose whose Mahalanobis Distance was larger than a specified threshold was deemed to contain outliers. In order to set the threshold, we used the fact that, since the Statistical Shape Model follows a multivariate Gaussian distribution $N\left({\varvec{x}}|{\varvec{\mu}}, \widetilde{C}\right)$, the squared Mahalanobis distance follows a chi-square distribution ${\chi }_{3Np}^{2}$^23^. Therefore, the confidence interval for outlier identification is automatically defined by the number of dimensions ${3N}_{p}$ and a chosen level of significance (e.g. $0.01$).

Second, whenever a pose was found to contain outliers, we identified the specific outlier body points by using an iterative procedure. We first excluded individual body points in order to obtain a set of $${N}_{p}$$ sub-poses, each containing ($${N}_{p}-1)$$ body points. We then re-calculated $$MD$$ for each sub-pose. The excluded body point associated with the smallest $$MD$$ was flagged as an outlier and removed. If the smallest $$MD$$ fell below the threshold the procedure was stopped. Otherwise, all the steps were repeated on the sub-pose associated with the removed outlier. During the iterative procedure described above, the confidence interval was automatically recalculated by adjusting the degrees of freedom for the $${\chi }^{2}$$ distribution according to the number of removed data points.

### Reconstruction of missing data

The last step of 3D-UPPER consists in re-estimating missing data. While a fraction of missing data is typically generated by deletion of outliers (see section above), additional missing data can already be present in the original dataset. Reconstruction of all missing data was performed by combining information from the Statistical Shape Model with the position of the inlier points according to the maximum likelihood principle.

In order to obtain a maximum likelihood estimate of the missing data we first decomposed the mean pose as ${{\varvec{\mu}}=[{\varvec{\mu}}}_{m} , {{\varvec{\mu}}}_{{\varvec{i}}{\varvec{n}}}]$ where ${{\varvec{\mu}}}_{m}$ indicates those elements associated with missing body points and ${{\varvec{\mu}}}_{in}$ those associated with inlier body points. Similarly, the covariance $\widetilde{C}$ can be decomposed as:

5 $$\widetilde{C}=\left[\begin{array}{cc}{\widetilde{C}}_{m,m}& {\widetilde{C}}_{m,in}\\ {\widetilde{C}}_{in.m}& {\widetilde{C}}_{in,in}\end{array}\right]$$

Therefore, the conditional distribution for the missing point coordinates ${{\varvec{x}}}_{{\varvec{m}}}$ can be expressed as:

6 $$p\left({{\varvec{x}}}_{m}\mid {{\varvec{x}}}_{in},{{\varvec{\mu}}}_{m},{{\varvec{\mu}}}_{in, } {\widetilde{C}}_{m,m}, {\widetilde{C}}_{in,m}\right) =\frac{1}{(2\pi {)}^\frac{3Nm}{2}{\left|{\widetilde{C}}_{m,m}\right|}^\frac{1}{2}}\mathrm{exp}\left\{-\frac{1}{2}{\left({{\varvec{x}}}_{m}-{{\varvec{\mu}}}_{m}-{\widetilde{C}}_{in,m}{\widetilde{C}}_{m,m}^{-1}\left({{\varvec{x}}}_{{\varvec{i}}{\varvec{n}}}-{{\varvec{\mu}}}_{{\varvec{i}}{\varvec{n}}}\right)\right)}^{T}{\left(\frac{\widetilde{C}}{{\widetilde{C}}_{m,m}}\right)}^{-1}\left({{\varvec{x}}}_{{\varvec{m}}}-{{\varvec{\mu}}}_{{\varvec{m}}}-{\widetilde{C}}_{in,m}{\widetilde{C}}_{m,m}^{-1}\left({{\varvec{x}}}_{{\varvec{i}}{\varvec{n}}}-{{\varvec{\mu}}}_{{\varvec{i}}{\varvec{n}}}\right)\right)\right\}$$

where ${{\varvec{x}}}_{{\varvec{i}}{\varvec{n}}}$ represent inlier body point coordinates while ${{\varvec{\mu}}}_{m}, {{\varvec{\mu}}}_{in},\boldsymbol{ }{\widetilde{C}}_{in,m}$ and ${\widetilde{C}}_{m,m}$ are provided by the Statistical Shape Model. Maximizing Eq. (6) in respect to ${{\varvec{x}}}_{{\varvec{m}}}$ leads to the estimation of the missing point coordinates as:

7 $${{\varvec{x}}}_{m}= {{\varvec{\mu}}}_{m}+{\widetilde{C}}_{in,m}{\widetilde{C}}_{in,in}^{-1}\left({{\varvec{x}}}_{{\varvec{i}}{\varvec{n}}}-{{\varvec{\mu}}}_{{\varvec{i}}{\varvec{n}}}\right)$$

### Validation of 3D-UPPER on simulated data

**Figure 2:**
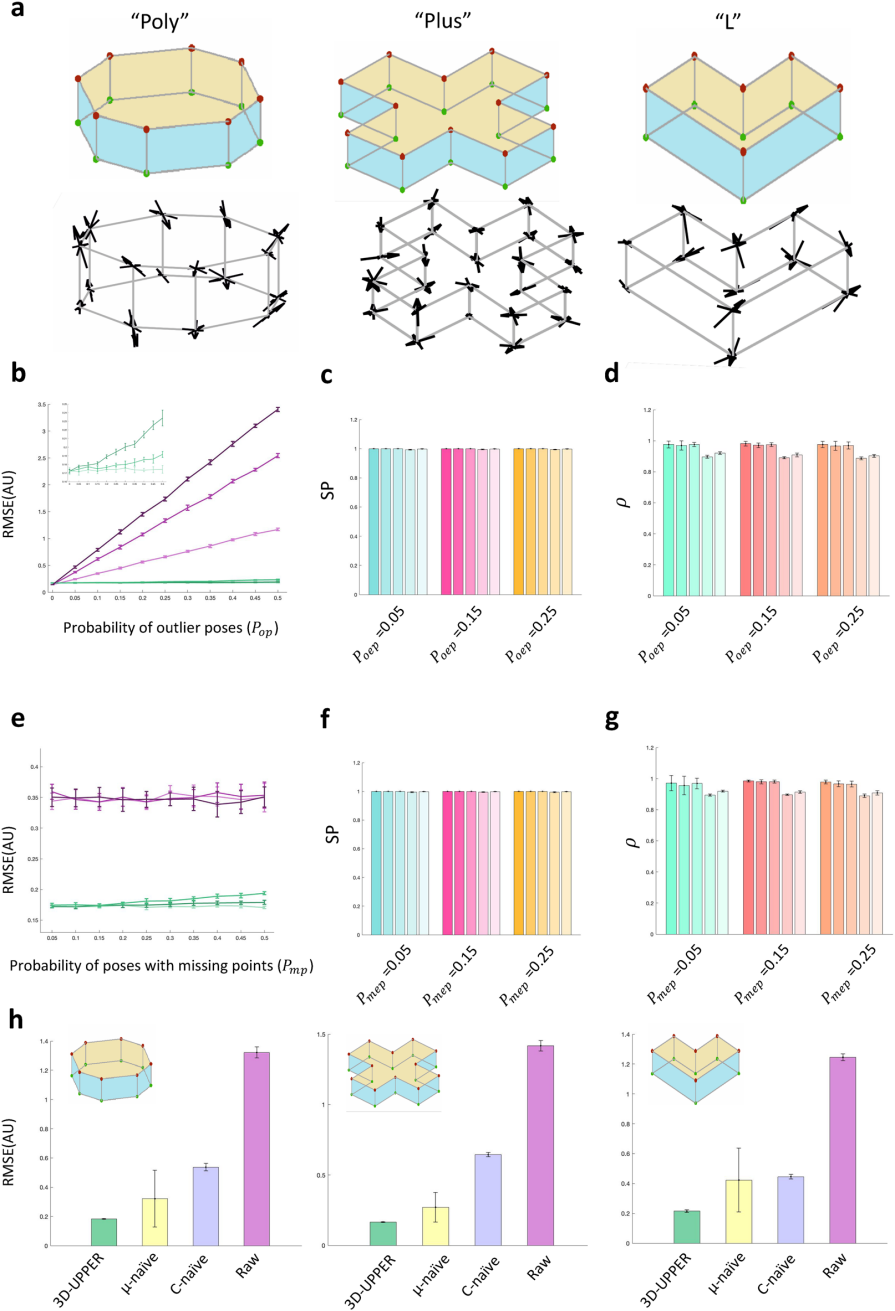
(**a**) Vertexes of three artificial shapes (top) and main directions of shape changes (bottom). These shapes, respectively called Poly, Plus and L, were used as simulated datasets for the validation of 3D-UPPER. (**b**) RMSE as function of ${P}_{op}$, the fraction of poses containing at least one outlier, for the Poly shape. Since shapes are simulated, the RMSE is reported in arbitrary units (AU). Purple lines indicate the RMSE between ground truth and contaminated data for different values of ${P}_{oep}$, the fraction of outliers within each contaminated pose (${P}_{oep}$ = 0.05, 0.15, 0.25 respectively for light, mid and dark lines). Green lines indicate the RMSE between ground truth and 3D coordinates recovered by using 3D-UPPER (${P}_{oep}$= 0.05, 0.15, 0.25 respectively for light, mid and dark lines). (**c**) Scalar product (mean ± SD) between the five ground-truth eigenposes and the eigenposes estimated with 3D-UPPER for the Poly shape (see ﻿also “Methods” for a definition of *SP*). Different shades are applied to enhance the visualization of the five eigenposes. Note that the scalar product is always close to unity irrespective of the value of ${P}_{oep}.$(**d**) Pearson’s correlation (mean ± SD) between shape parameters of ground truth [see Eq. (1)] and estimated with 3D-UPPER for the Poly shape (see ﻿also “Methods” for a definition of *ρ*). Different shades are applied to enhance the visualization of the five shape parameters. Correlations are close to unity irrespective of the value of ${P}_{oep}.$ (**e–g**) Same as panels (**b–d**) but here instead of outliers we introduce missing data, parametrized by ${P}_{mp},$ the fraction of poses containing at least one missing point, and ${P}_{mep}$, the fraction of missing points in each of such poses. Compared with panels (**b–d**), here we only show the reconstruction results obtained with 3D-UPPER, since RMSE cannot be computed for poses with missing data points. (**h**) Root-mean-square-error (mean ± SD) between ground truth data and 3D reconstructions obtained with 3D-UPPER and two alternative methods (µ-naïve and C-naïve). Left, middle and right panel indicates results for Poly, Plus and L shapes. For µ-naïve, the mean pose for the Statistical Shape Model is estimated simply by averaging aligned poses instead of using the algorithm described in “Robust estimation of the mean pose” section. For C-naïve, the eigenposes are estimated simply by the maximum-likelihood estimator instead of using the algorithm described in “Robust estimation of the eigenposes”. As comparison, we also report the RMSE between ground truth data and data contaminated according with the following parameters: ${P}_{oep}=0.15$, ${P}_{op}$ = 0.25, ${P}_{mep}$= 0.15, ${P}_{mp} =$ 0.25.

Our algorithm was first validated on simulated data in which we could systematically modify the number of meaningful changes in body shape (i.e. the eigenposes), the level of measurement noise, and the fraction of outliers and missing data. Since the algorithm can work with any type of body shape, we first tested it by simulating artificial objects. The mean poses were represented as vertices of such objects. To test the robustness of our algorithms across a range of different bodies we designed three different objects with increasing number of vertexes (Fig. 2a**,** top row; “Poly”, “Plus” and “L” objects represented respectively in left, middle and right panels). The shape of each object was then manipulated along simulated eigenposes (Fig. 2a**,** bottom row) as described in “Methods”.

We first tested the performance of 3D-UPPER by simulating a “ground-truth” dataset (based on the “Poly” shape) in which the object shape changed along 5 eigenposes. We then contaminated this dataset with outliers (see “Methods”). Increasing the fraction of poses containing outliers substantially increased the root mean square error (RMSE) distance between ground-truth and contaminated poses (Fig. 2b, purple error bars). However, the RMSE between ground-truth poses and poses recovered with 3D-UPPER was substantially reduced across all conditions (Fig. 2b**,** green error bars). Performance of 3D-UPPER was robust up to the condition in which 50% of the poses contained outliers, the worst condition we tested (Fig. 2b). Moreover, 3D-UPPER could reliably recover all the eigenposes (Fig. 2c) and the shape parameters associated with each pose (Fig. 2d).

Next, we tested the performance of 3D-UPPER by introducing missing body points. The RMSE between 3D-UPPER recovered poses and ground-truth data was substantially reduced in comparison to the RMSE calculated between contaminated and ground-truth data (Fig. 2e). Again, the performances were robust up to the condition in which 50% of the poses contained at least one missing body point. The presence of missing data points also did not affect the ability of the algorithm to recover eigenposes (Fig. 2f) and shape parameters (Fig. 2g).

Additional simulations were run to validate 3D-UPPER performance across a range of object parameters. We found such comparable performance was maintained across different simulated bodies (“Plus” shape: Supp. Fig. 1; “L” shape: Supp. Fig. 2) and for a different number of eigenposes (“Poly” shape: Supp. Fig. 3; “Plus” shape: Supp. Fig. 4; “L” shape: Supp.Fig. 5).

Finally, we wished to quantify the effect of our unsupervised Statistical Shape Model estimation on the ability of 3D-UPPER to recover the correct 3D coordinates. For each 3D object we simulated a dataset contaminated by measurement noise, outliers and missing data. We then used the contaminated data to estimate Statistical Shape Models. Estimation of mean pose ${\varvec{\mu}}$ and covariance matrix $\widetilde{C}$ for the Statistical Shape Models was performed (i) with the full set of 3D-UPPER estimators for ${\varvec{\mu}}$ and $\widetilde{C}$ (ii) with a naïve mean pose estimator and (iii) with a naïve covariance matrix estimator. We defined these models as “full”, “${\varvec{\mu}}$-naïve” and “$\widetilde{C}$-naïve” and we used them to reconstruct the 3D coordinates of the uncontaminated data (by applying Outlier Detection and Reconstruction of Missing Data as described above). The performance of each model was evaluated as Root Mean Square Error (RMSE) between uncontaminated and reconstructed data. We found that the full set of 3D-UPPER estimators returned more accurate reconstruction compared with “${\varvec{\mu}}$-naïve” and “$\widetilde{C}$-naïve” (Fig. 2h). These results indicate that both robust mean pose estimators and robust covariance estimators are necessary to match the aggregate 3D-UPPER performances.

### Validation of 3D-UPPER on data from 3D freely moving mice

We next tested 3D-UPPER performances on a real dataset of freely behaving mice (n = $4\times {10}^{5}$ frames from 7 animals). Landmarks were detected by using DeepLabCut^11^ from each individual camera from our four-camera system (see “Methods”). The 2D coordinates were then triangulated to generate an initial 3D reconstruction that we define as “raw 3D”. Finally, 3D-UPPER was applied to the raw 3D dataset to recover outliers and missing data.

This dataset was particularly challenging since the animals have a brain recording implant on the head that often occludes head or body landmarks from direct view of the cameras (see e.g. Fig. 1c,d). Therefore, we reasoned that a statistical shape model, endowed with knowledge of the geometrical relations between implant and head/body coordinates, could significantly improve our ability to estimate the occluded coordinates.

For this dataset we limited the number of eigenposes to five since those were sufficient to capture 90% variance in the full dataset (Supplementary Video 1). Visual inspection revealed that the first two eigenposes captured body arching and elongation along the main rostro-caudal axis. These shape changes are typically employed during rearing or at the onset of locomotion^14^. Instead, the third eigenpose capture left/right body torsions associated with orienting behaviours and freely moving exploration^14^.

We first asked whether the 3D-UPPER estimation of a Statistical Shape Model converged for a real 3D dataset contaminated by outliers and missing data. To do that we randomly resampled an increasingly larger set of poses from the full dataset (from 125 to 8000 poses). For each set size we calculated the scalar product of the eigenposes across 10 independently resampled sets. The expected value for this measure would be 1 for identical results across all sets and 0 when each set provides independent eigenposes.

**Figure 3:**
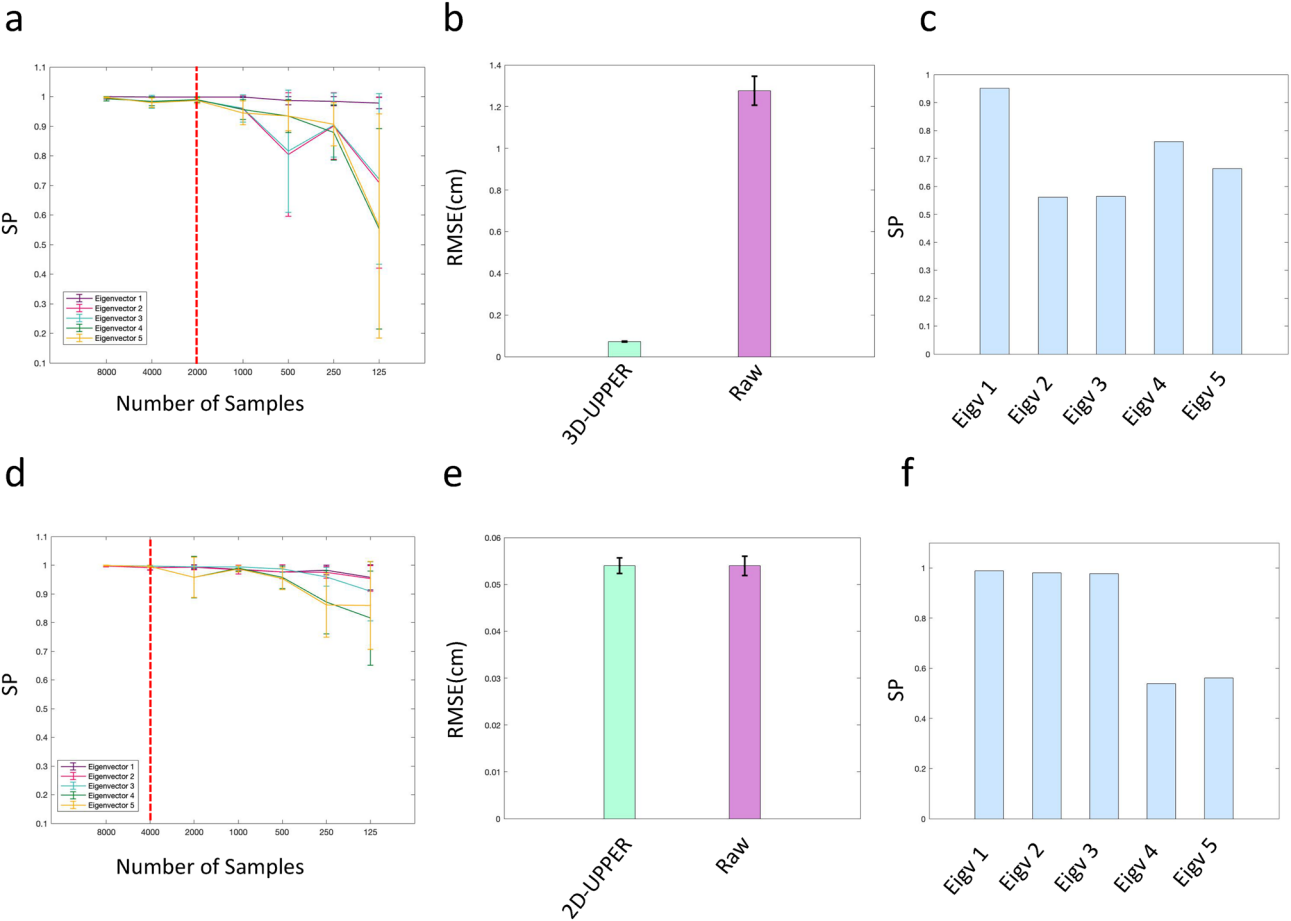
(**a**) Scalar product between eigenposes for independent samples of the dataset. If the eigenposes were the same across samples the scalar product would be equal to one. These results show that eigenposes estimation converges for samples larger than 2000 poses. (**b**) Root-mean-square-error (mean ± SD) between manually labelled data and data reconstructed with 3D-UPPER (green bar). As comparison we show the RMSE between manually labelled data and raw 3D reconstruction obtained by triangulating 2D data (purple bar). The 3D-UPPER algorithm, trained on raw 3D data, achieves a 17-fold reduction in RMSE. (**c**) Scalar product between the first five eigenposes estimated from 100 manually labelled poses with those obtained by using 3D-UPPER. Values inferior to one indicate that estimation of eigenposes based on few manually labelled poses can introduce bias. (**d–f**) Same as panels (**a–c**) but here we apply 2D-UPPER to a 2D dataset of freely moving mice.

We found that the estimation of eigenposes converged to 1 for samples larger than 2,000 poses (Fig. 3a). This result indicates that 3D-UPPER can capture invariant features of mouse body shape from any sufficiently large subset of data. Importantly, since convergence requires $\sim 2000$ poses, achieving this result by manually selecting uncontaminated poses would be highly time consuming.

We next asked whether 3D-UPPER could recover the correct 3D coordinates. Since for real data we do not have ground-truth values, we manually labelled a subset of poses (n = 100). We then compared the RMSE between manually labelled data and either raw 3D or 3D-UPPER reconstructed data. We found that 3D-UPPER substantially reduced RMSE compared with raw 3D data obtained simply by triangulating the 2D coordinates across the four recording cameras (Fig. 3b) consistently with previous results on simulated data. This result indicates that 3D-UPPER can use the Statistical Shape Model to the restore correct 3D coordinates of occluded body points (see Supplementary Video 2 for a representative example). We also found that the scalar products between the invariant 3D-UPPER eigenposes and the eigenposes obtained by the smaller set of manually labelled poses (n = 100) was substantially smaller than 1 (Fig. 3c). This confirms convergence results (Fig. 3a) indicating that manual annotation on small data samples can introduce bias in Statistical Shape Model estimation.

### Validation of 3D-UPPER on 2D freely moving data

Since our formulation of a Statistical Shape Model is based on vectorized poses the full algorithm can also be applied to 2D data with minimal modification. We refer to this version of the algorithm as 2D-UPPER. We tested 2D-UPPER performances on a different dataset of mouse behaviour recorded with a single overhead camera, since this is a standard set-up in many laboratories. The 2D dataset consisted of $6\times {10}^{4}$ poses from 15 animal.

We limited the number of eigenposes to 5 since those were sufficient to capture 90% variance in the full dataset. Like for the 3D dataset, the eigenposes captured behaviourally significant events. Thus, the first and third eigenpose captured respectively changes in body and head pitch typically associated with rearing, the second and the fifth captured left/right head and body torsions while the fourth eigenpose captured changes in body arching along the rostro-caudal axis (see Supplementary Video 3).

Similarly to what we found for 3D data, estimation of the eigenposes of a Statistical Shape Model converged for any data subset containing at least 4000 poses (Fig. 3d). Reconstruction performances were again assessed against a set of manually labelled poses (n = 100). We found that 2D-UPPER did not significantly reduced the RMSE (Fig. 3e), likely because the original tracking with DeepLabCut^11^ was already providing good results. This was confirmed by visual inspection of the tracked data. Finally, the first 3 eigenposes obtained by 2D-UPPER were well matched to those obtained from the smaller manually labelled dataset while two additional poses were less similar (Fig. 3f).

## Discussion

In order to understand natural behaviours, quantitative methods that accurately measure changes in body postures and movements are required. This is particularly important in the fields of experimental neuroscience and psychology, aiming to understand how the brain drives such behaviours. From those fields, in recent years a number of methods have been developed to track animal bodies from video recordings^5–7,10,11,24^. In spite of this progress, obtaining an accurate 3D reconstruction from freely moving animals still represents an open problem. To address this problem we developed the 3D-UPPER algorithm. Our main results show that, when applied to a real dataset of freely moving animals, 3D-UPPER can reduce by > 10-fold the 3D reconstruction error measured as Root Mean Square Error.

The traditional approach for performing 3D reconstruction relies on tracking 2D data from individual cameras followed by data triangulation. Therefore, the accuracy of the 3D reconstruction depends on accuracy of the 2D tracker. Body points that are occluded can generate outliers or missing 2D coordinates that substantially affect 3D reconstruction^14^. An assessment of 3D reconstruction accuracy can be performed by a human operator, but manual curation be can biased, as well as time consuming and unfeasible for large datasets. The alternative option is to develop a model that automatically assesses the 3D data based on the knowledge of the relations between body points. Statistical shape models, that have been widely used in medical imaging^16,17^ are ideal for this task. However, the possibility of using them for 3D reconstruction of animal behaviour had not been systematically established.

Our first contribution was to develop an algorithm to automatically estimate a statistical shape model (SSM). We designed the algorithm with the main aim of obtaining robust estimates of mean pose and eigenposes even from 3D data contaminated by large fractions of outliers and missing values. Therefore, compared to previous methods (see e.g.^14,17,18^), the algorithm we developed is unsupervised and does not require a manually annotated dataset. We applied 3D-UPPER on simulated data in which we could measure deviations from ground-truth coordinates. Our results showed that 3D-UPPER can recover ground-truth coordinates with high accuracy even when many poses (up to 50%) are contaminated by outliers and missing data. Such results were consistent for a variety of simulated objects of increased complexity. A robust unsupervised SSM estimation provided by 3D-UPPER was key to achieve these performances since naïve SSM estimators were substantially less accurate.

We then applied 3D-UPPER to a 3D dataset of freely moving mice. This dataset was particularly challenging since mice were carrying a head implant that occluded parts of the head and body from camera views. Previous studies on freely moving rodents used alternative tracking solutions based on accelerometers^25,26^ that however increase the weight on the animal head, or fluorescent markers^27^ that can be used for rats but not for mice. Unsupervised estimation of a Statistical Shape Model with 3D-UPPER converged rapidly and captured the same invariant eigenposes from any large enough sample of the full dataset (Fig. 3a). The SSM eigenposes captured meaningful actions involved in rearing and orienting behaviours. After SSM estimation we then used 3D-UPPER to recover outliers and missing data. We found that, compared with simple triangulation of 2D data, 3D-UPPER was able to reduce > 10-fold the error in 3D reconstruction. These results indicate that 3D-UPPER can accurately recover 3D poses in which occlusions generate outliers and missing data. Our current 3D data were acquired by using a wireless head-stage for brain recordings. The occurrence of occlusions could be higher for tethered systems in which the cables can also occlude camera views. An important future development will be to assess the performance of 3D-UPPER on such systems.

Other algorithms have been developed to perform 3D reconstruction of animal behaviour^28^. In the first instance, a simple post-hoc triangulation of 2D images can provide a good estimate of 3D body coordinates when body landmarks are not occluded in single camera views. However, occlusions occur systematically when animals are freely moving, introducing outliers and/or missing data. Increasing the number of cameras can improve 3D reconstructions however this comes at a substantial cost in terms of complexity of the experimental set-up^29^. To address this problem several software solutions have been developed. A common strategy has been to use a model that constrains the range of viable 3D coordinates according to spatial relationships among body parts^15,30^ and/or temporal relationships between consecutive frames. Alternatively, the recently developed DANNCE uses projective geometry to incorporate volumetric representations of animal bodies^10^. Finally, recent algorithms developed for 3D-lifting, i.e. to estimate 3D coordinates from individual camera views, have explicitly trained occlusion-aware networks^31–33^. Our 3D-UPPER algorithm also falls within the first class of algorithms. The advantage of 3D-UPPER relies in its simplicity: it is fully data driven and its implementation does not require any a priori knowledge about geometry of body shapes and constraints. Additionally, since the algorithm does not rely on temporal constraints (e.g. temporal smoothness over consecutive frames), can be applied to individual poses or static images. However, since its Statistical Shape Model is based on linear combinations, 3D-UPPER might not be able to fully capture nonlinear changes in body shapes. This simplification should be taken into account when analysing pathological behaviours that might deviate from our linear assumptions. Beyond such linearity constraints, it is also worth remarking that data imputation methods such as ours, that insert values derived from a model, are inherently biased against unexpected values. In spite of these caveats, our results on real 3D data indicate that 3D-UPPER can substantially outperform simple post-hoc triangulation by > 10-fold improvements in reconstruction accuracy.

Finally, we showed that our algorithm can also be employed on 2D data (2D-UPPER). Single overhead cameras still represent the standard set-up in many laboratories. Our results indicate that 2D-UPPER did not significantly improve reconstruction accuracy over that initially obtained with the state-of-art Deeplabcut tracking^5^. However, the associated Statistical Shape Model captured meaningful changes in body shape associated with rearing and orienting behaviours suggesting that 2D-UPPER could be useful for behavioural analysis.

## Methods

### Ethical statement

Experiments were conducted in accordance with the Animals, Scientific Procedures Act of 1986 (United Kingdom) and approved by the University of Manchester ethical review committee. All methods are reported in accordance with the ARRIVE guidelines^34^.

### Animals

The 2D and 3D data were collected respectively from 7 and 15 male, adult C57BL/6J mice (Charles River). All mice were initially kept in cages of 5 individuals. Animals were provided with food and water ad libitum throughout their life and kept on a 12:12 light dark cycle. Animals used to collect 3D data were housed individually after surgical implantation of the chronic electrodes. For a detailed description of the surgical procedure please refer to^35^. Animals used to collect 2D data were not implanted. During transfer between the cage and the behavioural arena we used the tube handling procedure instead of tail picking, as prescribed in^36^, in order to minimise stress and reduce variability across animals. At the end of the experiment animals were terminated by neck dislocation.

### Simulated data

The mean poses were designed as in (Fig. 2a). To generate the ${\varvec{P}}$ eigen-poses in our simulations, random vectors were first extracted from multivariate normal distribution and the vector basis was then made orthonormal by Gram-Smidt orthonormalization. To simulate changes in body position and orientation each pose was then independently rotated and translated.

In order to assess the performance of 3D-UPPER, additive noise in the 3D landmark positions and the fraction of missing data and of outliers were systematically varied.

Additive noise simulated the imperfection in tracking the exact 3D position in body landmark when the landmark was correctly labelled, but its spatial extent encompassed several pixels all representing reasonable solutions. We assumed additive noise to be zero mean, isotropic Gaussian and we parametrized it by its standard deviation ${\sigma }_{N}=0.1$ .

Missing data and outliers simulated gross errors in 3D data reconstruction. This typically happens when landmarks are occluded from direct view and their position can be hard to identify for a tracking software. In our simulation the ratio of outliers was systematically varied according to two parameters: the probability that a pose would contain at least one outlier (${p}_{op}$), and the mean fraction of outliers within each of such poses (${p}_{oep}$). Similar to additive noise, outliers were defined by their standard deviation ${\sigma }_{o}$ ,typically much larger than ${\sigma }_{N}$ . The occurrence of missing data was parametrized in the same and respectively by ${p}_{mp}$ and ${p}_{mep}$.

### 3D experimental data

Video footage was collected from 4 synchronized cameras placed over an open-field arena 30 cm × 30 cm, (see Fig. 1a and^37^). All data were acquired at 15 Hz by using Chamaleon 3 cameras (Point Grey). Body landmarks were separately tracked from each camera by using DeepLabCut. Training of DeepLabCut^11^ (version 2.1.6) was based on 1000 annotated images from a dataset of $4\times {10}^{5}$ images. Both training and inference were performed on an Ubuntu X machine by using a Titan RTX GPU (Nvidia, Santa Clara, California, USA). The raw 3D landmarks data were estimated by triangulation of the 2D coordinates as in^14^. Camera matrices were estimated by applying Direct Linear Transform to known object coordinates (Lego) as in^14^. All mice were implanted with chronic recording electrodes (Neuronexus; model: A4 × 4-3mm-50-125-177; package: CM16) and recorded with a wireless TBSI headstage and acquisition system.

### 2D experimental data

The 2D data were collected with a single overhead camera (same model used for the 3D) positioned over a large square arena (90 cm × 90 cm) which housed individual mice free during spontaneous exploration of the environment. All videos were sampled at 15 Hz. The mouse body points were labelled with DeepLabCut^11^ (version 2.1.6) after training the software over 200 frames from a dataset of ${6\times 10}^{4}$ images.

### Replacement of missing values with K-nearest neighbour

The K-nearest neighbour algorithm was used to fill up all missing values in each pose before estimating the covariance matrix. A target pose contains missing values was selected and the index of the missing values were defined as missing positions. Then, a set of recovering poses were aligned to a target pose. The set of recovering poses included all poses without missing values in the missing positions. Finally, the recovering poses were ranked according to their Euclidean distance with the target pose and the nearest K poses were averaged to replace the missing values in the target pose.

### Evaluation of 3D-UPPER performances

To evaluate performance of our algorithm we used three different measures: the Root-Mean-Square-Error $(RMSE)$ between ground-truth data with contaminated and reconstructed data in the 3D space; the scalar product ${SP=|p}_{j}{\widehat{p}}_{j}|$ between the ground-truth eigen poses ${[p}_{1, }\dots , {p}_{j} , \dots , {p}_{Nep, }]$ and those recovered by the UPPER methods ${[\widehat{p}}_{j, }\dots , {\widehat{p}}_{j} ,\dots , {\widehat{p}}_{Nep, }]$; the Pearson’s correlation ($\rho$) between original and reconstructed shape parameters ${b}_{ij}$ (see Eq. (1)). These measures quantified the ability of 3D-UPPER to minimise the average Euclidean distance between ground-truth and reconstructed poses (RMSE) and to capturing the relevant directions of changes in body shape over the whole dataset (SP) and the shape changes associated with each individual pose ($\rho )$.

## Supplementary Information

Supplementary Video 1.

Supplementary Video 2.

Supplementary Video 3.

Supplementary Figures.

## Data Availability

All the code for 3D-UPPER and 2D-UPPER has been written in MATLAB and it is available on GitHub (https://github.com/Aghileh/UPPER). The data used for this study are available on request from Riccardo Storchi (riccardo.storchi@manchester.ac.uk).
