# Supplementary material for: Three-dimensional unsupervised probabilistic pose reconstruction (3D-UPPER) for freely moving animals
Source: Sci Rep. 2023 Jan 4;13:155. doi: 10.1038/s41598-022-25087-4 (PMC9813182; doi:10.1038/s41598-022-25087-4)
Supplement: Supplementary file 4 — Supplementary Figures. [file 41598_2022_25087_MOESM4_ESM.docx]

Fig. S1. (**a**) RMSE as function of $P_{op}$, the fraction of poses containing at least one outlier, for the Plus shape. Purple lines indicates the RMSE between ground truth and contaminated data for different values of $P_{oep}$, the fraction of outliers within each contaminated pose ($P_{oep}$=0.05,0.15,0.25 respectively for light, mid and dark lines). Green lines indicate the RMSE between ground truth and 3D coordinates recovered by using 3D-UPPER ($P_{oep}$=0.05,0.15,0.25 respectively for light, mid and dark lines). (**b**) Pearson’s correlation (Mean±SD) between ground truth shape parameters (see equation 1) and shape parameters estimated with 3D-UPPER for the Poly shape. (**c**) Scalar product (Mean±SD) between the five ground truth eigenposes and the eigenposes estimated with 3D-UPPER for the Poly shape. Note that the scalar product is always close to unity irrespective of the value of $P_{oep}.$Correlations are close to unity irrespective of the value of $P_{oep}.$ (**d-f**) Same as panels (**b-d**) but here instead of outliers we introduce missing data, parametrized by $P_{mp},$the fraction of poses containing at least one missing point, and $P_{mep}$, the fraction of missing points in each of such poses.

Fig. S2. Same as Fig. S1 but for L shape.

Fig. S3. Same as Fig.S1 but for Poly shape. In this simulation we only introduced 3 eigenposes in the ground truth data compared with data shown in Fig. 2b-g were we simulated 5 eigenposes. This is reliably reflected in scalar product of eigenposes (panel c & f) that is ~1 for the first 3 eigenposes but lower for the 4^th^ and 5^th^ eigenpose.

Fig. S4. Same as Fig. S3 but for Plus shape.

Fig. S5. Same Fig. S3 but for L shape

**Supplementary Video legends**

Supplementary Video 1: This video shows an animation of the first five eigenposes which were sufficient to capture 90% variance in the full 3D dataset of free moving animal. The first two eigenposes captures body arching and elongation along the main rostro-caudal axis, which typically happened during rearing or at the onset of locomotion. The third eigenpose captures left/right body torsions associated with orienting behaviours and freely moving exploration. The fourth and fifth eigenposes show more subtle adjustments in body shape.

Supplementary Video 2: This representative video demonstrates the result of 3D-UPPER reconstruction on freely moving animal. Left panels show two camera views while right panels show 3D reconstruction from a side and top view.

Supplementary Video 3: This video shows the first five eigenposes which were sufficient to capture 90% of the variance in the 2D dataset. The first and third eigenposes capture respectively changes in body and head pitch, typically associated with rearing. The second and the fifth eigenposes capture left/right head and body torsions. The fourth eigenpose captures changes in body arching along the rostro-caudal axis.
